# Two novel bornaviruses identified in colubrid and viperid snakes

**DOI:** 10.1007/s00705-021-05138-3

**Published:** 2021-06-15

**Authors:** Florian Pfaff, Dennis Rubbenstroth

**Affiliations:** Institute of Diagnostic Virology, Friedrich-Loeffler-Institut, 17493 Greifswald, Riems Germany

## Abstract

We present the complete genome sequences of Caribbean watersnake bornavirus (CWBV) and Mexican black-tailed rattlesnake bornavirus (MRBV), which we identified in archived raw transcriptomic read data of a Caribbean watersnake (*Tretanorhinus variabilis*) and a Mexican black-tailed rattlesnake (*Crotalus molossus nigrescens*), respectively. The genomes of CWBV and MRBV have a length of about 8,900 nucleotides and comprise the complete coding regions and the untranslated regions. The overall genomic makeup and predicted gene content is typical for members of the genus *Orthobornavirus* within the family *Bornaviridae*. Alternative splicing was detected for the L and M genes. Based on a phylogenetic analysis of all viral proteins, we consider both viruses to be members of a single novel species within the genus *Orthobornavirus*. Both viruses form a distinct outgroup to all currently known orthobornaviruses. Based on the novel virus genomes, we furthermore identified closely related endogenous bornavirus-like nucleoprotein sequences in transcriptomic data of veiled chameleons (*Chamaeleo calyptratus*) and a common lancehead (*Bothrops atrox*).

The family *Bornaviridae* belongs to the order *Mononegavirales* [1] and comprises viruses with a monopartite single-stranded RNA(-) genome that form enveloped and spherical virions with a diameter of 70-130 nm. Their genomes are about 8.9 kb in length and encode the nucleoprotein (N), the accessory protein X, the phosphoprotein (P), the matrix protein (M), the glycoprotein (G), and the large protein (L), which contains the RNA-directed RNA polymerase. The organization of the respective open reading frames varies among the different bornaviruses, and additional protein isotypes produced by alternative splicing [2] or start codon skipping [3] have been reported. Bornaviruses are divided into the three taxonomic genera: *Orthobornavirus*, *Carbovirus*, and *Cultervirus* [1, 4].

Currently, orthobornaviruses have the widest known host spectrum within the family *Bornaviridae*, ranging from mammals and birds to reptiles, whereas carbo- and culterviruses have so far only been identified in reptiles [5, 6] and fish [7], respectively. Mammalian orthobornaviruses are known to be zoonotic agents and may be transmitted from reservoirs, such as shrews or squirrels, to humans, sheep and horses [8–10]. Reptilian carbo- and orthobornaviruses have so far been identified in Australian carpet pythons (*Morelia spilota* (Lacépède, 1804)) with neurological disease [5] and in a wild-caught Loveridge's garter snake (*Elapsoidea loveridgei* (Parker, 1949)) [6], respectively. Furthermore, partial sequences of exogenous orthobornavirus-like N, X, and P genes identified in a Gaboon viper (*Bitis gabonica* (Duméril, Bibron & Duméril, 1854)) [11] suggested a wider distribution of orthobornaviruses among snakes.

In this study, we used data mining of transcriptomic and metagenomic raw RNA read archives in order to identify hitherto undetected bornaviruses of reptiles. As a result, we determined the full genome sequence of two bornaviruses in datasets from colubrid and viperid snakes.

In detail, we initially employed the Serratus website [12] in order to identify datasets within the Sequence Read Archive (SRA) that potentially contain bornavirus-like sequences. We then downloaded promising SRA datasets (SRR5440420 and SRR9693197), trimmed them with respect to quality and adapter contamination using Trim Galore (v0.6.6), and subsequently used rnaSPAdes (v3.13.0) for *de novo* assembly. The resulting contigs were then screened for bornavirus-like sequences using DIAMOND BLASTX (v0.9.21.122) with a representative database of bornavirus proteins. For both SRA datasets, the initial assembly yielded a single contig representing the complete viral genome. These initial contigs were then further polished using an iterative mapping and assembly strategy [13]. The full genomes were annotated with respect to known bornaviruses using Geneious Prime (v2021.0.1). Furthermore, we predicted introns and splice sites using STAR (v2.7.7a) running in basic two-pass mode. The splice sites deduced from raw reads were further evaluated by *in silico* prediction using NNSPLICE (v0.9). Transcription termination sites were predicted using sequence similarity [14] and manual inspection of raw reads showing transition to polyA at the respective termination position.

SRR5440420 contains raw reads from the Harderian gland transcriptome of a wild-caught adult Caribbean watersnake (*Tretanorhinus variabilis* (Duméril, Bibron & Duméril, 1854); family Colubridae) from Santa Fe, La Habana, Cuba [15]. SRR9693197 contains raw reads from the venom gland transcriptome of a wild-caught juvenile Mexican black-tailed rattlesnake (*Crotalus molossus nigrescens* (Gloyd, 1936); family Viperidae) from Nuevo León, Mexico. The bornaviruses identified and characterised in these datasets were named Caribbean watersnake bornavirus (CWBV) and Mexican black-tailed rattlesnake bornavirus (MRBV), respectively. The genomes of CWBV and MRBV are of similar length, with MRBV (8907 nt) being three nucleotides longer at the 5’ end than CWBV (8904 nt). The overall genomic makeup of the two viruses is very similar.

In detail, we predicted six protein coding open reading frames, encoding N, X, P, M, G, and L (Fig. 1). The gene order N-X/P-M-G-L is consistent with the genome organization of members of the genus *Orthobornavirus* but different from that of members of the genera *Carbovirus* and *Cultervirus*, which share the order N-X/P-G-M-L [5, 7]. Furthermore, we identified three conserved transcription initiation sites and four transcription termination sites, as well as alternative splicing for the M and L genes (Fig. 1). A third potential intron, located in the L gene, was identified only for MRBV. All predicted splicing events were supported by several reads missing the intron sequence in both datasets. All predicted transcription start sites (S1-3) correlated well with a steep increase in read coverage, while all predicted transcription termination sites (T1-4) correlated with an steep drop in read coverage and reads showing a transition to polyA at the respective termination site. When compared to representative orthobornaviruses, the 5’ and 3’ untranslated region of CWBV and MRBV can be considered complete, although further experiments need to be performed using molecular methods such as rapid amplification of cDNA ends (RACE) PCR.

**Fig. 1:**
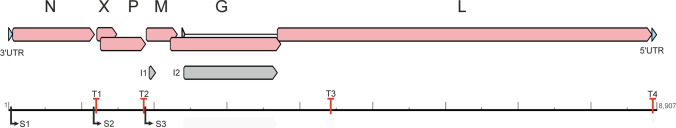
The overall genome organization of Caribbean watersnake bornavirus (CWBV) and Mexican black-tailed rattlesnake bornavirus (MRBV) is highly conserved and comprises the canonical bornaviral gene order N-X/P-M-G-L (pink arrows) flanked by untranslated regions (UTR – blue arrows). Introns were detected for the M and L genes (grey arrows). Three transcription initiation sites (S1-3) and four transcription termination sites (T1-4) were predicted.

A phylogenetic analysis of a concatenated alignment of N-P-M-G-L amino acid sequences from both viruses along with representative bornavirus sequences showed that both viruses were distantly related to other members of the genus *Orthobornavirus* (Fig. 2a). Pairwise sequence comparison (PASC [16]) of complete genome sequences revealed 76.6% pairwise nucleotide sequence identity between CWBV and MRBV and 56.6-57.3% identity to the most closely related orthobornaviruses. Based on phylogenetic analysis and the conserved genome organisation, and in line with the species demarcation cutoff of 72 to 75% pairwise nucleotide sequence identity [1], we suggest that both viruses be assigned to a single new species within the genus *Orthobornavirus.*

**Fig. 2:**
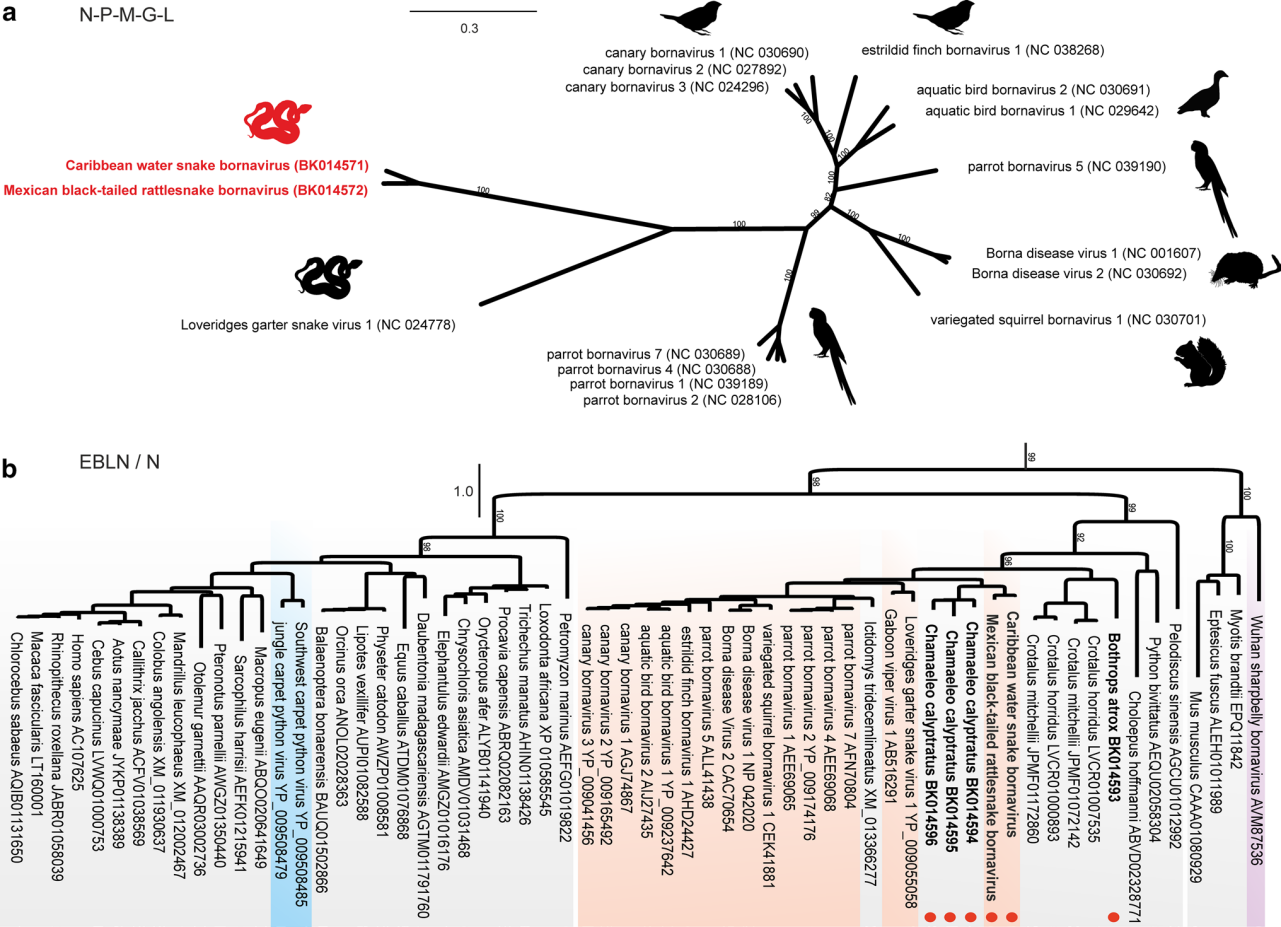
Maximum-likelihood phylogenetic analysis of orthobornaviruses (a) and endogenous bornavirus-like nucleoproteins (b). (a) An unrooted phylogenetic tree based on the concatenated amino acid sequence alignments of N-P-M-G-L of the novel snake bornaviruses (highlighted in red) together with all available complete genome sequences of members of the genus *Orthobornavirus*. (b) Phylogenetic relationship between endogenous bornavirus-like nucleoprotein amino acid sequences (grey) and those of members of the bornavirus genera *Carbovirus* (blue), *Orthobornavirus* (orange) and *Cultervirus* (purple). Sequences determined in this study are indicated by red dots. Trees were calculated using IQ-TREE (version 2.1.2; 1 million ultrafast bootstraps; optimal substitution model for each alignment/alignment partition). The bars represent amino acid substitutions per site, and numbers in italics indicate bootstrap support for the major branches.

Finally, we used the CWBV and MRBV protein sequences to further search the SRA. For this purpose, we downloaded 2-8 million reads of all available transcriptomics or metagenomics datasets related to members of the taxonomic order Squamata and used DIAMOND BLASTX to match sequences. Datasets with promising hits were then assembled as described. As a result, we identified three endogenous bornavirus-like nucleoprotein (EBLN) sequence elements in veiled chameleons (*Chamaeleo calyptratus* (Duméril & Bibron, 1851)*;* SRR6662597) [17] and one EBLN in a common lancehead (*Bothrops atrox* (Linnaeus, 1758); SRR1953004) [18] (Fig. 2b). The chameleon EBLNs exhibited 68.8-75.1% pairwise nucleotide sequence identity to each other and 65.2-66.3% to the CWBV and MRBV N genes, whereas the common lancehead EBLN was more distantly related. A phylogenetic comparison of EBLNs and circulating bornaviruses based on the frameshift-corrected protein alignment of Hyndman et al. [5] showed that a common ancestor of both novel bornaviruses left its genetic fingerprint in the genomes of non-avian reptiles. Currently, there is no evidence that these viruses cause any disease in the sampled snakes, and further screening is needed to evaluate their distribution and clinical relevance. However, these sequences will improve bornavirus diagnostic procedures and help researchers to understand the evolutionary origins of these viruses.

## Data Availability

The annotated genome sequences generated during and/or analysed during the current study are available in the DDBJ/EMBL/GenBank databases under the TPA accession numbers BK014571, BK014572, BK014593-BK014596. Additional metadata and raw sequencing data are available under the BioProject ID PRJNA382075 (SAMN06706898, SRR5440420) and PRJNA554814 (SAMN12284706, SRR9693197).
